# Passively versus Actively Detected Malaria: Similar Genetic Diversity but Different Complexity of Infection

**DOI:** 10.4269/ajtmh.17-0364

**Published:** 2017-09-05

**Authors:** Zuleima Pava, Irene Handayuni, Leily Trianty, Retno A. S. Utami, Yusrifar K. Tirta, Agatha M. Puspitasari, Faustina Burdam, Enny Kenangalem, Grennady Wirjanata, Steven Kho, Hidayat Trimarsanto, Nicholas Anstey, Jeanne Rini Poespoprodjo, Rintis Noviyanti, Ric N. Price, Jutta Marfurt, Sarah Auburn

**Affiliations:** 1 Global and Tropical Health Division, Menzies School of Health Research, Charles Darwin University, Darwin, Northern Territory, Australia;; 2 Eijkman Institute for Molecular Biology, Jakarta, Indonesia;; 3 Mimika District Health Authority, Timika, Papua, Indonesia;; 4 Timika Malaria Research Programme, Papuan Health and Community Development Foundation, Timika, Papua, Indonesia;; 5 Maternal and Child Health and Reproductive Health, Department of Public Health, Faculty of Medicine, Universitas Gadjah Mada, Yogyakarta, Indonesia;; 6 Centre for Tropical Medicine and Global Health, Nuffield Department of Clinical Medicine, University of Oxford, Oxford, United Kingdom

## Abstract

The surveillance of malaria is generally undertaken on the assumption that samples passively collected at health facilities are comparable to or representative of the broader *Plasmodium* reservoir circulating in the community. Further characterization and comparability of the hidden asymptomatic parasite reservoir are needed to inform on the potential impact of sampling bias. This study explores the impact of sampling strategy on molecular surveillance by comparing the genetic make-up of *Plasmodium falciparum* and *Plasmodium vivax* isolates collected by passive versus active case detection. Sympatric isolates of *P. falciparum* and *P. vivax* were collected from a large community survey and ongoing clinical surveillance studies undertaken in the hypomesoendemic setting of Mimika District (Papua, Indonesia). *Plasmodium falciparum* isolates were genotyped at nine microsatellite loci and *P. vivax* at eight loci. Measures of diversity and differentiation were used to compare different patient and parasitological sample groups. The results demonstrated that passively detected cases (symptomatic) had comparable population diversity to those circulating in the community (asymptomatic) in both species. In addition, asymptomatic patent infections were as diverse as subpatent infections. However, a significant difference in multiplicity of infection (MOI) and percentage of polyclonal infections was observed between actively and passively detected *P. vivax* cases (mean MOI: 1.7 ± 0.7 versus 1.4 ± 1.4, respectively; *P* = 0.001). The study findings infer that, in hypomesoendemic settings, passive sampling is appropriate for molecular parasite surveillance strategies using the predominant clone in any given infection; however, the findings suggest caution when analyzing complexity of infection. Further evaluation is required in other endemic settings.

## BACKGROUND

Calls for the global elimination of malaria have renewed political interest in public health interventions, reigniting control activities and placing elimination back on the agenda for many endemic countries.^1^ With the wide-scale deployment of key intervention strategies such as early diagnosis, prompt treatment, and intense vector control, 33 countries have reached the malaria elimination stage between 2000 and 2015.^2^ However, as the incidence of malaria falls in endemic settings, increasingly sensitive diagnostic tools are revealing large reservoirs of low-density malaria infections highlighting a far greater ‘depth’ of malaria burden.^3–6^ These subpatent infections are not routinely detectable by conventional microscopy and can persist for several months without clinical manifestations. Both of the predominant species, *P. falciparum* and *P. vivax*, are able to cause submicroscopic infections, although the relative proportion of subpatent cases tends to be higher in *P. vivax* than *P. falciparum*.^2,7^

Subpatent infections can be detected in a wide range of malaria-endemic settings, with the proportion tending to rise in low transmission areas.^7–9^ As countries approach malaria elimination, it is crucial to identify every infection to stop ongoing transmission and prevent resurgence. In this context, intense surveillance is required to monitor clinical and parasitological changes that can inform the most effective intervention strategies.

The majority of such surveillance efforts have been undertaken at health facilities, recruiting symptomatic individuals with patent parasitaemia seeking treatment (“passive case detection”). As malaria elimination efforts progress, more in-depth knowledge of the parasite population in asymptomatic community cases is needed, as this reservoir contributes a greater proportion of the total parasite burden. An important question is whether the genetic make-up of actively detected parasites is adequately represented by those detected passively, or whether the different sampling strategies can result in a significant bias which can affect the interpretation of a given study. The surveillance of antimalarial drug resistance could be particularly vulnerable to sampling bias, because phenotypic and molecular studies are generally applied to clinical isolates with little focus given to the asymptomatic population. In the case of ex vivo drug susceptibility testing, stringent criteria are applied which can result in even more constrained sampling.^10,11^ Furthermore, in pre-elimination settings, the low number of malaria cases in parasite population genetic studies often requires pooling of samples from patients with different disease presentations, collected using different sampling strategies and methodologies. These studies are being used increasingly to inform local trends in parasite transmission as reflected by measures of within-host and population diversity.^12,13^ A recent population genetic study of *P. falciparum* in a low endemic area of Zambia inferred that actively and passively detected infections formed distinct subpopulations with differing levels of diversity, but the study was partly constrained by the difference in years of collection between the actively and passively detected cases.^14^ Another study conducted in the higher endemic setting of Mimika District, Papua, Indonesia, using samples from both clinical and community cases revealed a subpopulation of *P. falciparum* isolates only observed in community cases.^15^ However, the latter study did not directly investigate the level of genetic differentiation or differences in population diversity and infection complexity between the actively and passively detected *P. falciparum* isolates. Furthermore, to date, no studies have directly compared the genetic make-up between actively and passively detected *P. vivax* infections, which contribute a large burden of malaria infections in the Asia-Pacific region.

This study used sympatric *P. falciparum* and *P. vivax* isolates obtained from a community survey and ongoing clinical surveillance studies in Mimika District, Papua, Indonesia, to compare the genetic make-up of samples collected by passive versus active case detection, and between patent and subpatent groups.

## MATERIALS AND METHODS

### Study site and sampling.

#### Passive case detection.

The study was performed in Mimika District, Papua, Indonesia. Nearly 90% of the population of approximately 182,000 people live in lowland areas where unstable malaria transmission occurs. Although all four species of human-only infecting *Plasmodium* parasites can be found in the area, *P. falciparum* and *P. vivax* are the most common species causing 41% and 49% of malaria monospecies infections, respectively. *Plasmodium malariae* accounts for approximately 4% of malaria cases and less than 1% of cases are caused by *P. ovale*. Mixed-species malaria infections can be found in up to 6% of cases.^16^ Rumah Sakit Mitra Masyarakat (RSMM) is the main hospital in the district. RSMM is located in the capital city, Timika, and serves approximately 150,000 people, assessing up to 300 patients/day, 6 days/week. Patients with uncomplicated malaria due to any *Plasmodium* species are treated with dihydroartemisinin-piperaquine as per the national first-line protocol.^17^

Samples at the RSMM were collected within the framework of two studies. Between March 2011 and August 2014, patients presenting with an uncomplicated *P. vivax* or *P. falciparum* monoinfection, a peripheral parasitaemia between 1,000 μL^−1^ and 80,000 μL^−1^, as determined by microscopic examination, and more than 70% of ring stage parasites were invited to participate in an ongoing ex vivo antimalarial susceptibility surveillance study (ex vivo susceptibility study, ESS). Between March 2015 and February 2016, patients presenting with uncomplicated *P. falciparum* or *P. vivax* malaria with peripheral parasitaemia greater than 1,000 μL^−1^ and 250 μL^−1^, respectively, but with no restriction by parasite stage, were invited to participate in an ongoing treatment efficacy study (TES). After obtaining written consent, 5 mL of venous blood was collected for all patients by venipuncture and filtered using cellulose columns to deplete the host white blood cells.^18^ The postfiltration packed infected red blood cells were used for DNA extraction.

#### Active case detection.

Active case detection was undertaken within the framework of a cross-sectional survey conducted in Timika between April and July 2013. Details on the sampling methods of the survey can be found elsewhere.^16^ Briefly, 800 houses were selected by cluster randomized sampling. First, the four largest subdistricts were chosen purposively. Consecutively, the number of clusters per district was calculated according to their respective relative population. Finally, 20 houses were chosen randomly within each cluster, following World Health Organization recommendations. Individuals living in the same house and residing in the study area for at least 6 months were included in the study. Symptomatic patients were defined as any individual with a current fever episode (i.e., axillary temperature > 37.5°C) or in the last 24 hours. Venous blood from one adult volunteer and 200 µL of capillary blood in a Microtainer™ from the remaining members of each household were collected and used for blood film examination, hemoglobin measurement, and molecular analysis.

### DNA extraction and species confirmation.

*Plasmodium* species was assessed initially by microscopy using Giemsa-stained thick and thin blood films. Peripheral parasitaemia was determined from the number of parasites per 200 white blood cells, assuming a white cell count of 7,300 cells/µL. Asexual stage composition was determined by classifying 100 parasites into ring, trophozoite, or schizont stage on examination of a thick smear.

For actively detected cases, genomic DNA (gDNA) was extracted from 50 µL packed red blood cell (RBC) pellets using the QIAamp 96 DNA Blood Kit (Qiagen, Chadstone, Victoria, Australia). For passively detected cases, 2 mL of packed RBCs were extracted using the QIAamp DNA Midi Kit (Qiagen, Chadstone, Victoria, Australia). *Plasmodium* species confirmation was undertaken in duplicate with 2 µL gDNA template using a nested PCR protocol as described elsewhere.^19^ *Plasmodium falciparum*, *P. vivax*, *P. malariae*, and *P. ovale* small-subunit rRNA DNA clones (MRA-177, MRA-178, MRA-179, and MRA-180; ATCC, Manassas, VA) were used as positive controls.

### Microsatellite typing.

*Plasmodium falciparum* genotyping was conducted using nine previously described short tandem repeat (STR) markers (ARAII, *Pf*PK2, poly-alpha, TA1, TA42, TA60, TA81, TA87, and TA109), following the protocol described by Anderson et al.^20^ For *P. vivax* genotyping, nine previously described STR markers selected as a consensus panel by the Asia Pacific Malaria Elimination Network Vivax Working Group (*Pv3.27*, MS16, MS1, MS5, MS8, MS10, MS12, MS20, and msp1F3) were used following previously described protocols.^21,22^ The fluorescently labeled PCR products were separated by capillary electrophoresis on an ABI 3100 Genetic Analyser with GeneScan LIZ-600 size standard (Applied Biosystems, Mulgrave, Victoria, Australia). The resulting electrophoretograms were analyzed using VivaxGEN version 1.0 and verified manually.^23^ To reduce potential artifacts from background noise, an arbitrary fluorescent intensity threshold of 50 relative fluorescence units was used. Only samples with information in at least 50% of the loci were considered as successfully genotyped.

### Data analysis.

#### Population diversity and genetic differentiation.

Diversity was estimated by means of allelic richness (*Rs*). *Rs* was calculated by quantifying the number of alleles and normalizing the value to the sample size, using the hierfstat package in R.^24^ The expected heterozygosity (*H*_E_), which is defined as the probability of finding a different allele at a specific locus when randomly selecting a pair of samples from the same population, was measured as an additional index of population diversity. The pairwise *F*_ST_ metric was used to determine the genetic distance between populations and interpreted according to the classification of Balloux and Lugon-Moulin^25^ (i.e., low if the *F*_ST_ value was less than 0.05; moderate if between 0.05 and 0.15; high if between 0.15 and 0.25; and very high if greater than 0.25). Arlequin software (version 3.5) was used to calculate *H*_E_, *F*_ST_, and the standardized measure (*F*’_ST_), which adjusts for high marker diversity. Significance values for the *F*_ST_ were derived using 10,000 permutations of the data in Arlequin.

#### Linkage disequilibrium (LD).

To assess changes in transmission over time and to further characterize the parasites constituting the different subgroups, multilocus LD was measured using the standardized index of association (*I*_*A*_^*S*^). First, multilocus genotypes (MLGs) were reconstructed from the predominant allele at each locus in isolates with no missing data. Then, using the MLGs, *I*_*A*_^*S*^ was measured using the web-based LIAN 3.5 software, assessing the significance of the estimates using 10,000 permutations of the data.^26^ The analysis was also performed using low-complexity infections (maximum of one multiallelic locus) and with each unique MLG represented just once.

#### Polyclonality and multiplicity of infection.

Malaria parasites are haploid during the asexual stage; hence, the presence of more than one allele at a given locus in an individual sample is indicative of multiple parasite clones. In contrast to single nucleotide polymorphism-based markers, which would only reach up to four variants, the abundance of alleles at STR markers greatly facilitates the detection of polyclonal infections. However, the quality and quantity of parasite DNA may impact the sensitivity to detect minor alleles in polyclonal infections. In theory, minor clones are more likely to be identified in samples with larger quantities of parasite DNA. For this reason, Anderson and colleagues introduced the application of a minimum relative peak intensity threshold for calling minor alleles to enhance the comparability of multiplicity of infection (MOI) and polyclonality estimates between parasite populations with differing DNA quality or quantities.^20^ In view of the different sample qualities and quantities evaluated in the current study, minor alleles were only called if their height was at least 33% of the major allele’s height: a threshold that has been applied in multiple previous population genetic studies of malaria.^27^ An infection was defined as polyclonal if more than one allele was observed at any locus. The MOI for a given sample was defined as the maximum number of alleles at any locus. The number of polyclonal loci per sample was also estimated for closer inspection of the complexity of individual infections in the effort to identify any subtle differences between groups.

#### Statistical tests.

R and SPSS software were used for statistical analysis with a significance level at *P* < 0.05.^28^ Subgroup analysis was performed according to 1) patient recruitment method (active or passive) and 2) infection patency (i.e., patent or subpatent) using the χ^2^ test and Fisher’s Exact Test. Differences in MOI and *H*_E_ between subgroups and species were assessed using the Mann–Whitney U or Kruskal–Wallis test.

#### Ethics approval.

Ethical approval for this study has been obtained from the Eijkman Institute Research Ethics Commission, Eijkman Institute for Molecular Biology, Jakarta, Indonesia (EIREC-47, EIREC-67, and EIREC-75), and the Human Research Ethics Committee of the Northern Territory Department of Health & Families and Menzies School of Health Research, Darwin, Australia (HREC 2010-1396).

## RESULTS

### Marker properties.

A summary of the diversity and genotyping success rate for each of the *P. falciparum* and *P. vivax* markers in all genotyped samples is presented in Supplemental Table 1. Moderate to high diversity was found for all *P. falciparum* markers except for TA42 and TA109, particularly in the TES and ESS samples (Supplemental Table 1). By contrast, high diversity was found in all *P. vivax* markers across all sample sets. However, the MS8 assay presented artifacts that affected the reliability of the allele calling and this locus was, therefore, excluded from further analysis. Genotype information for 50% of the marker set (five loci for *P. falciparum* and four loci for *P. vivax*) was obtained from 84.5% (414/492) *P. falciparum* samples and from 70.7% (390/552) of *P. vivax* samples. Most of the failed samples were asymptomatic *P. vivax* or *P. falciparum* infections (97%; 157/162 and 99%; 77/78, respectively).

### Sampling groups.

#### Passively detected cases.

All passively detected cases presented with microscopically confirmed parasitaemia. The comparability of the isolates collected from the clinical and ex vivo studies were assessed before pooling. No difference was observed between the median parasitaemia of the TES and ESS samples either in *P. falciparum* (*N* = 45, median_TES_ 12,240 parasites μL^−1^; and *N* = 94, median_ESS_ 37,143 parasites μL^−1^; *P* = 0.145) or *P. vivax* (*N* = 46, median_TES_ 11,041 parasites μL^−1^; and *N* = 104, median_ESS_ 16,224 parasites μL^−1^; *P* = 0.306). Furthermore, given that the TES and ESS samples were collected across multiple years, an assessment of temporal differences in MOI, polyclonality, LD, *H*_E_, and *Rs* was undertaken with grouping by year of collection. Over the 6 years of sample collection, the proportion of polyclonal infections for *P. falciparum* varied from 0% to 31%, (*P* = 0.085) and from 23% to 67% (*P =* 0.104) for *P. vivax,* whilst the mean MOI ranged from 1.0 to 1.3 for *P. falciparum*, and from 1.2 to 2.0 for *P. vivax* (*P* = 0.085 and *P* = 0.104, respectively; Supplemental Table 2). Population diversity (*H*_E_) values ranged from 0.448 to 0.608 for *P. falciparum* (*P* = 0.648) and from 0.840 to 0.881 for *P. vivax* (*P* = 0.932). Furthermore, there was no difference between *Rs* for *P. falciparum* (*R*_*S*_ = 2.571–3.328; *P* = 0.333) or *P. vivax* (*R*_*S*_ = 4.927–5.481; *P* = 0.429). LD demonstrated modest fluctuations between the study years, with a slight increase observed in the *P. falciparum* cases in 2015 (Supplemental Table 3). Nonetheless, because there were no significant differences in diversity or complexity of infection between the study years, all TES and ESS samples were pooled into a single group defined as the passively detected patent sample group (PP) for subsequent analyses.

#### Actively detected cases.

Only 18 of the isolates collected by active case detection were symptomatic (*N* = 5 *P. vivax* and *N* = 13 *P. falciparum*). The small sample size did not allow comparisons with other subgroups and, therefore, these were excluded from further analyses. Because venous blood samples were only taken from adults, MOI, polyclonality, *Rs*, and *H*_E_ were compared between adults (≥ 15 years) and children (< 15 years), but no significant differences were observed in any of these measures (Supplemental Table 4). Likewise, there were no significant differences in MOI, polyclonality, *Rs*, and *H*_E_ between isolates from capillary or venous samples (Supplemental Table 5). Samples were, therefore, pooled into the following two subgroups: actively detected patent infections (AP) and actively detected subpatent infections (AS). In summary, AP comprises asymptomatic cases with a slide positive for *P. falciparum* or *P. vivax* monoinfection, whereas AS comprises asymptomatic cases with a slide negative for malaria, but PCR positive for either *P. falciparum* or *P. vivax* monoinfection.

The demographic details of all subgroups are presented in Table 1. Similar characteristics were observed across the groups in all features other than parasitaemia. The overall median parasitaemia was similar between the two species (Mann–Whitney U Test, *P* = 0.482), but significantly different between active and passively detected cases for both species (*P* < 0.0001).

**Table 1:** Demographic data of all cases

Subgroups	Age,* *N* (%)			Males *N* (%)	Parasitaemia† median (range)	Total
	< 5	5–15	> 15			
*P. falciparum*^13^						
* *Passively detected‡	0	19 (13.6)	121 (86.4)	68 (48.6)	14,837 (3,280–361,728)	140
* *Actively detected patent§	9 (8.6)	31 (29.5)	65 (61.9)	49 (46.7)	600 (27–132,256)	105
* *Actively detected subpatent‖	9 (4.1)	57 (26.3)	151 (69.6)	81 (37.3)	n/a	217
All cases	18 (4.4)^2^	107 (23.1)^5^	337 (72.9)^6^	204 (42.9)	7,595 (27–361,728)	4,6213
*P. vivax*^5^						
* *Passively detected‡	0	28 (18.2)	126 (81.8)	75 (48.4)	15,072 (1,640–120,576)	155
* *Actively detected patent§	20 (23.3)	25 (29.1)	41 (47.7)	40 (46.5)	385 (38–19,200)	86
* *Actively detected subpatent‖	17 (7.4)	72 (31.4)	140 (61.1)	90 (39.3)	n/a	229
All cases	37 (8.0)^1^	125 (26.4)	311 (65.6)^4^	207 (43.6)	7,884 (38–120,576)	4,755

Superscript numbers indicate excluded symptomatic community survey samples.

* Age in years.

† Parasites/µL.

‡ Symptomatic patent infections.

§ Asymptomatic patent infections.

‖ Asymptomatic subpatent infections.

### Stage composition and within-host diversity.

Isolates for ex vivo susceptibility testing (ESS) were only selected if the ring stage composition was ≥ 70% and thus, the potential confounding of parasite staging was assessed. After excluding the ESS and AS samples, *P. falciparum* infections almost all comprised predominantly ring stages in both actively detected patent (AP; 89.4%, 84/94) and passively detected cases (PP, 97.8%, 44/45; *P* = 0.104). By contrast, the percentage of synchronous ring stage *P. vivax* infections was 11.1% (9/81) in AP compared with 41.3% (19/46) in PP samples (*P* > 0.001; Supplemental Table 6).

Among the AP cases, *P. falciparum* infections showed similar complexity between synchronous (MOI_S_ = 1.23; SD ± 0.4) and asynchronous infections (MOI_A_ = 1.30; SD ± 0.5; *P* = 0.604), with polyclonal infections present in 23% (19/84) and 30% (3/10) of the cases, respectively (*P* = 0.694). Similarly, *P. vivax* infections showed comparable complexity of infection between synchronous and asynchronous infections: MOI_S_ = 1.78 (SD ± 1.1) versus MOI_A_ = 1.75 (SD ± 0.8; *P* = 0.820), as well as proportion of polyclonal infections: 44% (4/9) and 54% (39/72), respectively (*P* = 0.728). Likewise, comparison between synchronous and asynchronous passively detected *P. vivax* cases (ESS and TES) revealed no significant difference in complexity of infection (MOI_S_ = 1.39 (±0.6) versus MOI_A_ = 1.52 (±0.8); *P* = 0.463), or percentage of polyclonal infections (34% (42/123) versus 41% (11/27), *P* = 0.516; Table 2). Because there was only one asynchronous *P. falciparum* infection among the passively detected cases, no further comparisons could be performed. However, when the three datasets were combined, the results remained similar (Table 2).

**Table 2:** Within-host diversity and ring stage synchronicity in *Plasmodium falciparum and Plasmodium vivax* sample sets

Sample sets	Synchronous*	*N* (%)	MOI Mean (SD)†	*P*‡	Polyclonality *N* (%)	*P*§
*P. falciparum*						
* *Actively detected cases‖^1^	Yes	84 (89)	1.23 (0.42)	0.604	19 (23)	0.694
	No	10 (11)	1.30 (0.48)		3 (30)	
* *Passively detected cases¶	Yes	138 (99)	1.12 (0.33)	–	17 (12)	–
	No	1 (1)	–		–	
*P. vivax*						
* *Actively detected cases‖^6^	Yes	9 (11)	1.78 (1.09)	0.820	4 (44)	0.728#
	No	72 (89)	1.75 (0.82)		39 (54)	
* *Passively detected cases¶^5^	Yes	123 (82)	1.39 (0.60)	0.463	42 (34)	0.516
	No	27 (18)	1.52 (0.75)		11 (41)	

MOI = multiplicity of infection.

* Ring stage >70%.

† Standard deviation.

‡ Mann–Whitney *U* test.

§ χ^2^.

‖ Community survey.

¶Treatment efficacy study and ex vivo surveillance study.

# Fisher’s exact test.

** Superscript indicates number of cases with missing data.

### Parasite density and within-host diversity.

Assessment of MOI and parasitaemia in patent infections revealed a weak, but significantly negative correlation between MOI and parasitaemia for *P. vivax* (rho = −0.400, *P* = 0.032), but not for *P. falciparum* (rho = −0.095, *P* = 0.146). However, the correlation was not apparent after excluding the ESS samples for neither *P. vivax* (rho = −0.117, *P* = 0.184), nor *P. falciparum* (rho = −0.133, *P* = 0.112).

### Population diversity, population differentiation, and LD analysis.

There was no significant difference in the *P. falciparum Rs* between AP and AS (*P* = 0.635), between AP and PP (*P* = 0.170), and between PP and AS (*P* = 0.095; Table 3). Similarly, *P. vivax Rs* was comparable between AP and AS (*P* = 0.640), between AP and PP (*P* = 0.809), and between PP and AS (*P* = 0.799; Table 3). *H*_*E*_ values were also comparable across the subgroups in both species (Table 3). *F*_ST_ values varied from 0.007 to 0.047 and −0.002 to 0.003 for *P. falciparum* and *P. vivax*, respectively. Likewise, no differentiation between subgroups was observed for *P. falciparum* or *P. vivax* infections (Table 4).

**Table 3:** Within host and genetic diversity analysis of *Plasmodium falciparum* and *Plasmodium vivax* subgroups

Subgroups	*N*	MOI Mean* (±SD)	Polyclonal infections *N* (%)	*R*_*S*_† Mean (±SD)
*P. falciparum*				
Passively detected§	139	1.1 (0.329)	17 (12)	5.771 (2.81)
Actively detected patent‖	100	1.2 (0.423)	23 (23)	6.476 (2.06)
Actively detected subpatent	164	1.2 (0.393)	27 (16)	6.392 (1.95)
All actively detected	264	1.2 (0.405)	50 (19)	6.731 (2.08)
*P. vivax*				
Passively detected	150	1.4 (0.626)	53 (35)	17.790 (7.20)
Actively detected patent	82	1.7 (0.843)	43 (52)	14.645 (7.72)
Actively detected subpatent	155	1.6 (0.686)	78 (50)	14.654 (6.75)
All actively detected	237	1.7 (0.745)	121 (51)	16.540 (8.80)

MOI = multiplicity of infection.

* Standard deviation.

† Allelic richness.

‡ Symptomatic patent infections

§ Asymptomatic patent infections.

‖ Asymptomatic subpatent infections.

**Table 4:** Pairwise differentiation between *Plasmodium falciparum* and *Plasmodium vivax* subgroups

*P. falciparum*	Passively detected*	Actively detected patent†	Actively detected subpatent‡
Passively detected*	*	0.0532	0.1191
Actively detected patent†	0.02287**	*	0.021
Actively detected subpatent‡	0.04713***	0.00707^NS^	*
*P. vivax*			
Passively detected*	*	0.0131	0.0205
Actively detected patent†	0.00194^NS^	*	−0.0100
Actively detected subpatent‡	0.00318^NS^	−0.00154^NS^	*

*F*_*ST*_ (*P* value) in lower left triangle. *F’*_*ST*_ in upper right triangle. NS = not significant; *P* > 0.05 = *; *P* > 0.001 = **; *P* > 0.0001 = ***.

* Symptomatic patent infections.

† Asymptomatic patent infections.

‡ Asymptomatic subpatent infections.

There was no evidence of significant LD in any of the *P. vivax* subgroups, with *I*_A_^S^ scores ranging from 0 to 0.008 (Table 5). By contrast, significant LD was observed in each of the *P. falciparum* subgroups, with higher *I*_A_^S^ scores, ranging from 0.05 to 0.15 (Table 5). The highest levels of LD were observed in the actively detected *P. falciparum* subgroups. Further analysis using unique MLGs did not reveal evidence of clonal expansion in any of the subgroups in either species (Table 5).

**Table 5:** Linkage disequilibrium between actively detected patent infections (AP), actively detected subpatent infections (AS), and passively detected patent infections (PP)

Subgroup	All infections N	*I*_*A*_^*S*^	Low complexity	*I*_*A*_^*S*^	Unique MLGs N	*I*_*A*_^*S*^
			N			
*P. vivax*						
Passively detected*	150	0.0062^NS^	123	0.0043^NS^	119	0.0038^NS^
Actively detected patent†	82	0.008^NS^	49	−0.0001^NS^	73	0.008^NS^
Actively detected subpatent‡	155	−0.0067^NS^	107	−0.01^NS^	67	−0.0067^NS^
*P. falciparum*						
Passively detected*	139	0.0542***	129	0.0543***	95	0.02***
Actively detected patent†	100	0.1354***	158	0.1325***	62	0.12***
Actively detected subpatent‡	164	0.1462***	85	0.146***	71	0.13***

Only samples with no missing data were included in the analyses. *** *P* < 0.001; NS = not significant.

* Symptomatic patent infections.

† Asymptomatic patent infections.

‡ Asymptomatic subpatent infections.

### Within-host diversity: complexity of infection and polyclonality.

As summarized in Table 3, the mean MOI and the proportion of polyclonal infections was similar between AP and AS infections in *P. falciparum* (MOI_AP_ = 1.2 (±0.42), 23%; and MOI_SP_ = 1.2 (±0.39), 16%; *P* = 0.199 and *P* = 0.189, respectively). Similar MOI and polyclonality rates were also observed between AP and AS infections in *P. vivax* (MOI_AP_ = 1.7 [±0.84], 52%; and MOI_AS_ = 1.6 [±0.69], 50%; *P* = 0.407 and *P* = 0.721, respectively).

Although actively detected *P. falciparum* cases showed a higher mean MOI and a higher proportion of polyclonal infections compared with the passively detected infections, the difference was only statistically significant between the proportion of polyclonal infections in the AP (23%) compared with the PP cases (12%; *P* = 0.035; Table 3). By contrast, when comparing *P. vivax* subgroups, the actively detected cases showed consistently higher mean MOI (MOI_AP_ = 1.7, [±0.84], MOI_AS_ = 1.6, [±0.69]) compared with the passively detected infections (MOI_PP_ = 1.4, [SD ± 0.63], *P* = 0.003), and a higher proportion of polyclonal infections (51% and 35%, respectively, *P* = 0.003; Table 3).

**Figure 1.**
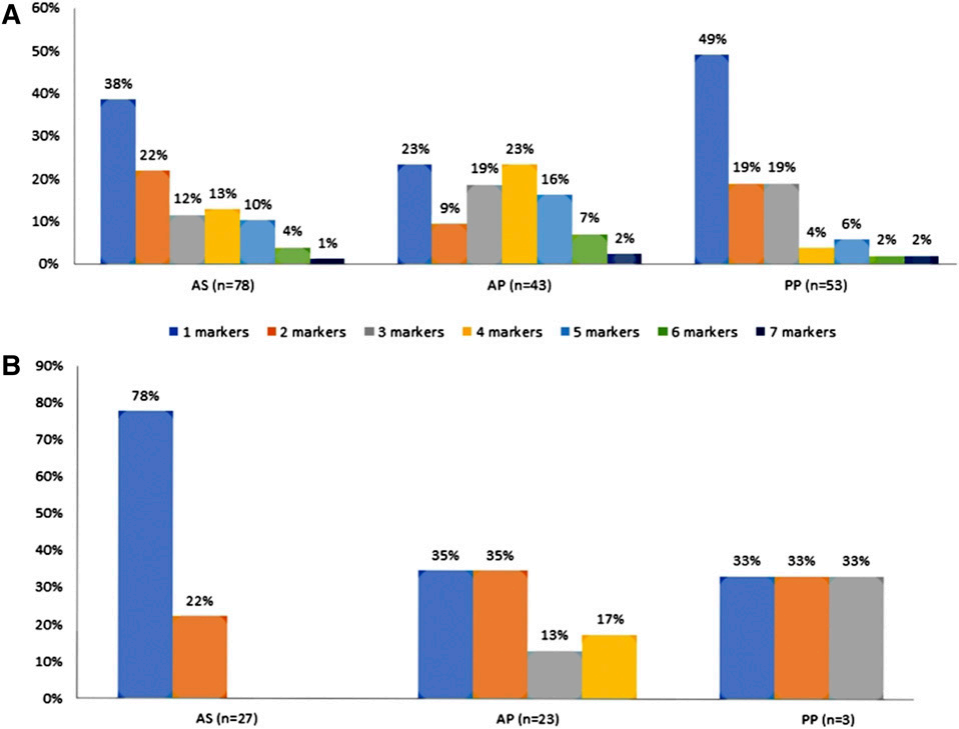
Polyclonal loci between asymptomatic subpatent infections (AS), asymptomatic patent infections (AP), and symptomatic patent infections (PP) in (**A**) *Plasmodium vivax* and (**B**) *Plasmodium falciparum.* This figure appears in color at www.ajtmh.org.

The number of multiallelic loci (MLOCI) in the polyclonal infections in each of the *P. vivax* and *P. falciparum* subgroups is illustrated in Figure 1. The actively detected polyclonal *P. vivax* infections presented a higher average MLOCI than those detected passively (MLOCI_A_ = 2.8, [±1.7], MLOCI_P_ = 2.1, [±1.5]; *P* = 0.010). The actively detected *P. falciparum* infections presented a lower average MLOCI than passively detected infections but the results were not statistically significant (MLOCI_A_ = 1.6, [±0.9], MLOCI_P_ = 2.0, [±1.1]; *P* = 0.140).

## DISCUSSION

This study presents a comprehensive comparative genetic analysis of actively versus passively detected cases of *P. falciparum* and *P. vivax*. Knowledge regarding potential sampling bias is vital for the effective design and interpretation of surveillance studies of malaria, particularly passive sampling strategies. The current study highlights the challenges that are associated with the interpretation of molecular data generated from different sampling sources and the key issues to consider for sampling strategies.

In a previous study conducted in Mimika district, genetic substructure was observed in the *P. falciparum* population, with a small subpopulation of infections found in actively detected cases only.^15^ On further investigation of the genetic differences between *Plasmodium* populations collected by active versus passive sampling strategies conducted in the same population, the current study revealed that there was no evidence of any significant differences in genetic diversity between the passively and actively detected subgroups in either *P. falciparum* or *P. vivax*. Furthermore, low differentiation between the subgroups in both *P. falciparum* and *P. vivax* confirmed that the different subgroups appear to comprise the same general pool of parasite strains. The highest levels of genetic differentiation were observed in comparisons of actively versus passively detected cases in *P. falciparum* (*F*_ST_ = 0.023–0.05; *F’*_ST_ = 0.05–0.12), likely reflecting the aforementioned small subpopulation of asymptomatic infections and possibly also inflated by modest temporal changes in LD. Indeed, LD analysis revealed higher allelic association among the actively detected *P. falciparum* cases in comparison to the passively detected cases. Nonetheless, the *F*_ST_ and *F’*_ST_ values observed between the *P. falciparum* subgroups were all markedly lower than those reported by Noviyanti et al.^29^ between different Indonesian islands (*F*_ST_ = 0.08–0.41; *F’*_ST_ = 0.23–0.78).

The equivalence in population diversity and low differentiation between the ESS and the other sample subgroups suggests that despite the application of strict sample selection,^10,11^ the results of drug susceptibility testing are likely to be representative of the parasites circulating in the broader population. Therefore, if parasites with resistance-conferring mutations were arising in the community, they would be likely to be detected in the ex vivo surveillance study.

The results of the study have important implications for our understanding of the molecular epidemiology of the parasites beneath the surface of the “malaria-iceberg.” This is especially relevant in the context of mass drug administration campaigns, as one of the greatest concerns of this practice is the selection of resistant parasites circulating in the community at very low frequencies.^30^ Our results suggest that passive surveillance studies are a reliable “index” of the parasite strains circulating in the broader population. However, direct comparison of known resistance genotypes in patent versus subpatent infections will be required to confirm these findings.^31^

Our study also suggests that parasites derived from passively detected symptomatic infections had similar genotypes to actively detected asymptomatic infections. These findings suggest that the development of clinical symptoms may be more attributable to host factors such as immunity and RBC polymorphisms, than to intrinsic parasite factors resulting in a higher multiplication rate. Similar results were reported by Ferreira et al.^32^ who explored the genetic background of *P. falciparum* parasites in cases of uncomplicated versus cerebral malaria, finding no evidence of closer relatedness (i.e., no evidence of strain specificity) among the cerebral cases. In a genetic study of *P. falciparum* and *P. vivax* in Colombia, Pacheco et al.^33^ also found no evidence of differences in the circulating genotypes between severe and uncomplicated cases in either species. However, further studies need to be performed to support this hypothesis.

These observations are particularly relevant when considering the sampling strategy for population genetic studies of *P. falciparum* and *P. vivax*. The similar estimates of diversity and low differentiation in comparisons of different blood withdrawal methods (capillary versus venous samples), disease presentation (symptomatic/passively detected versus asymptomatic/actively detected), and concentration of DNA (patent versus subpatent) in both species, indicate that diversity estimates are not affected by any of these conditions. Therefore, population diversity and other population genetic measures derived from predominant allele calls can be reliably estimated on pooled samples and effectively compared between different sample types.

Population genetic studies routinely use estimates of complexity of infection as indicators of transmission intensity.^34^ In contrast to the population-level estimates of diversity, which are based on frequencies of the major alleles in a given infection, assessments of the deeper complexity within infections revealed several differences between sampling groups. Actively detected cases exhibited a higher MOI than passively detected cases of both species, albeit only reaching statistical significance in *P. vivax*, but not in *P. falciparum*. This finding has important implications for sampling strategies in population genetic studies of *P. vivax* investigating within-host diversity. The contrast between the species could reflect interspecies differences in the persistence of subpatent infections resulting from new infections or relapses. In a recent longitudinal survey of individuals with asymptomatic malaria parasitaemia in a low endemic setting in Cambodia where *P. vivax* predominates, Tripura et al.^35^ showed that 35% of individuals with *P. vivax* infection remained parasitaemic for up to 11 months, whereas only 13% of individuals with *P. falciparum* infection remained parasitaemic for up to 4 months. The persistence of asymptomatic parasitaemia might enhance the opportunity for multiple inoculations of *P. vivax* by different mosquitoes before the onset of clinical symptoms or parasite clearance.

Several previous studies have highlighted the challenges of characterizing multiple-clone infections using STR typing.^27,36^ These challenges mainly relate to the presence of stutter peaks or nonspecific PCR products that can interfere with the discrimination of artifacts from true minor alleles. Moreover, higher DNA concentrations can increase the detection of minor alleles.^20,36,37^ To counter potential differences in sample quality or quantity in population genetic studies of *Plasmodium*, minimum threshold values of 25% or 33% for calling additional alleles relative to the major allele are commonly applied.^20,27,36^ The negative correlation between parasitaemia and MOI in the current study suggests that applying a relative threshold such as 33% is not as effective in *P. vivax* samples as it was in *P. falciparum* samples when pooling samples from the community with those attending health facilities. Consequently, these results suggest caution when inferring transmission intensity from MOI and percentage of polyclonal infections from pooled samples.

The results of our study differ from those reported by Searle et al.^14^ who found that actively detected *P. falciparum* infections collected in a low endemic setting in Zambia were significantly divergent from passively detected cases, not only in terms of complexity of infection, but also in their genetic background. Key differences between the two studies that may explain the observed discrepancy are the greater temporal heterogeneity in sampling of active versus passively detected cases combined with greater bottlenecking in the *P. falciparum* population in the Zambian study. The Searle et al.^14^ study shows evidence of increasingly unstable *P. falciparum* transmission over time, including increased inbreeding, consistent with rapidly declining transmission. These temporal genetic changes could account for the differences found between the beginning of the sampling period (when most of the actively detected cases were enrolled) and the end of the sampling period (when most of the passively detected cases were enrolled) as observed in other temporal genetic studies of *P. falciparum*.^38,39^ By contrast, the current study comprises samples collected in a period during which there was no marked change in malaria transmission and, with the actively detected cases enrolled within the sampling period of the passively detected cases. Differing from the situation in *P. falciparum*, reports on *P. vivax* population diversity remaining relatively resilient to changes in the intensity of transmission suggest that,^12,34^ except in areas with extreme bottlenecking such as Malaysia,^40^ passively detected cases should remain representative of the broader *P. vivax* population.

A limitation of this study is the large proportion of adult males missed from sampling during the cross-sectional study, which constitutes a bias per se that needs to be considered. The study also presents a potential bias reflecting the omission of very low-density infections that exhibited low genotyping success. Furthermore, the recently described high-volume, ultrasensitive PCR approach by Imwong et al.^3^ may have identified parasites at an even lower level of the “malaria-iceberg” that were not identified with the conventional PCR approach used here. However, it is also highly likely that these very low-density infections would be refractory to STR genotyping.^3^ Lastly, given the multiple factors affecting the dynamics of asymptomatic malaria and the influence of malaria transmission on parasite diversity and parasite differentiation, the results of the current study may not necessarily be extrapolated to areas with different malaria epidemiology.^41,42^ Indeed, available evidence inferred that the minor subpopulation of asymptomatic *P. falciparum* infections observed in Mimika district might reflect infections imported from other parts of Indonesia.^16^ As local transmission declines, the impact of imported cases on the parasite population diversity and structure may become more pronounced.^40^

In conclusion, asymptomatic malaria infections constitute a major challenge in pre-elimination areas where their prevalence may force the need to modify ongoing case detection strategies for surveillance. Many studies continue to use passively detected samples as an index of diversity and drug resistance in the broader parasite population. Our results suggest that passively detected samples provide effective representation of the ‘strains’ circulating in the broader community including the hidden asymptomatic reservoir. However, passive sampling strategies may not be effective for investigation of within-host infection complexity in the asymptomatic reservoir. These results highlight critical considerations when undertaking molecular surveillance of malaria.

## Supplementary Material

Supplemental Tables.

## References

[1] Feachem RG Sabot OJ, 2007 Global malaria control in the 21st century: a historic but fleeting opportunity. JAMA 297: 2281–2284. 17519417 10.1001/jama.297.20.2281

[2] WHO, 2016 *Eliminating Malaria* Geneva, Switzerland: WHO, 24.

[3] Imwong M Hanchana S Malleret B Renia L Day NP Dondorp A Nosten F Snounou G White NJ, 2014 High-throughput ultrasensitive molecular techniques for quantifying low-density malaria parasitemias. J Clin Microbiol 52: 3303–3309. 24989601 10.1128/JCM.01057-14 PMC4313154

[4] Phommasone K 2016 Asymptomatic *Plasmodium* infections in 18 villages of southern Savannakhet Province, Lao PDR (Laos). Malar J 15: 296. 27234446 10.1186/s12936-016-1336-0 PMC4882819

[5] Waltmann A 2015 High rates of asymptomatic, sub-microscopic *Plasmodium vivax* infection and disappearing *Plasmodium falciparum* malaria in an area of low transmission in Solomon Islands. PLoS Negl Trop Dis 9: e0003758. 25996619 10.1371/journal.pntd.0003758 PMC4440702

[6] Britton S Cheng Q McCarthy JS, 2016 Novel molecular diagnostic tools for malaria elimination: a review of options from the point of view of high-throughput and applicability in resource limited settings. Malar J 15: 88. 26879936 10.1186/s12936-016-1158-0 PMC4754967

[7] Cheng Q Cunningham J Gatton ML, 2015 Systematic review of sub-microscopic *P. vivax* infections: prevalence and determining factors. PLoS Negl Trop Dis 9: e3413. 25569135 10.1371/journal.pntd.0003413 PMC4288718

[8] Bousema T Okell L Felger I Drakeley C, 2014 Asymptomatic malaria infections: detectability, transmissibility and public health relevance. Nat Rev Microbiol 12: 833–840. 25329408 10.1038/nrmicro3364

[9] Wu L van den Hoogen LL Slater H Walker PG Ghani AC Drakeley CJ Okell LC, 2015 Comparison of diagnostics for the detection of asymptomatic *Plasmodium falciparum* infections to inform control and elimination strategies. Nature 528: S86–S93. 26633770 10.1038/nature16039

[10] Russell B 2008 Determinants of in vitro drug susceptibility testing of *Plasmodium vivax*. Antimicrob Agents Chemother 52: 1040–1045. 18180357 10.1128/AAC.01334-07 PMC2258486

[11] Kerlin DH Boyce K Marfurt J Simpson JA Kenangalem E Cheng Q Price RN Gatton ML, 2012 An analytical method for assessing stage-specific drug activity in *Plasmodium vivax* malaria: implications for ex vivo drug susceptibility testing. PLoS Negl Trop Dis 6: e1772. 22880143 10.1371/journal.pntd.0001772 PMC3413709

[12] Barry AE Waltmann A Koepfli C Barnadas C Mueller I, 2015 Uncovering the transmission dynamics of *Plasmodium vivax* using population genetics. Pathog Glob Health 109: 142–152. 25891915 10.1179/2047773215Y.0000000012 PMC4455355

[13] Arnot D, 1998 Unstable malaria in Sudan: the influence of the dry season. Clone multiplicity of *Plasmodium falciparum* infections in individuals exposed to variable levels of disease transmission. Trans R Soc Trop Med Hyg 92: 580–585. 10326095 10.1016/s0035-9203(98)90773-8

[14] Searle KM; Southern Africa International Centers of Excellence for Malaria Research, 2017 Distinct parasite populations infect individuals identified through passive and active case detection in a region of declining malaria transmission in southern Zambia. Malar J 16: 154. 28420399 10.1186/s12936-017-1810-3 PMC5395854

[15] Pava Z 2017 Genetic micro-epidemiology of malaria in Papua Indonesia: extensive *P. vivax* diversity and a distinct subpopulation of asymptomatic *P. falciparum* infections. PLoS One 12: e0177445. 28498860 10.1371/journal.pone.0177445 PMC5428948

[16] Pava Z 2016 Submicroscopic and asymptomatic *Plasmodium* parasitaemia associated with significant risk of anaemia in Papua, Indonesia. PLoS One 11: e0165340. 27788243 10.1371/journal.pone.0165340 PMC5082812

[17] Elyazar IR Hay SI Baird JK, 2011 Malaria distribution, prevalence, drug resistance and control in Indonesia. Adv Parasitol 74: 41–175. 21295677 10.1016/B978-0-12-385897-9.00002-1 PMC3075886

[18] Auburn S 2013 Effective preparation of *Plasmodium vivax* field isolates for high-throughput whole genome sequencing. PLoS One 8: e53160. 23308154 10.1371/journal.pone.0053160 PMC3537768

[19] Singh B Bobogare A Cox-Singh J Snounou G Abdullah MS Rahman HA, 1999 A genus- and species-specific nested polymerase chain reaction malaria detection assay for epidemiologic studies. Am J Trop Med Hyg 60: 687–692. 10348249 10.4269/ajtmh.1999.60.687

[20] Anderson TJ Su XZ Bockarie M Lagog M Day KP, 1999 Twelve microsatellite markers for characterization of *Plasmodium falciparum* from finger-prick blood samples. Parasitology 119: 113–125. 10466118 10.1017/s0031182099004552

[21] Karunaweera ND Ferreira MU Munasinghe A Barnwell JW Collins WE King CL Kawamoto F Hartl DL Wirth DF, 2008 Extensive microsatellite diversity in the human malaria parasite *Plasmodium vivax*. Gene 410: 105–112. 18226474 10.1016/j.gene.2007.11.022

[22] Koepfli C Mueller I Marfurt J Goroti M Sie A Oa O Genton B Beck HP Felger I, 2009 Evaluation of *Plasmodium vivax* genotyping markers for molecular monitoring in clinical trials. J Infect Dis 199: 1074–1080. 19275476 10.1086/597303

[23] Trimarsanto H 2017 VivaxGEN: an open access platform for comparative analysis of short tandem repeat genotyping data in *Plasmodium vivax* populations. PLoS Negl Trop Dis 11: e0005465. 28362818 10.1371/journal.pntd.0005465 PMC5389845

[24] Goudet J, 2005 HIERFSTAT, a package for R to compute and test hierarchical F-statistics. Mol Ecol Notes 5: 184–186.

[25] Balloux F Lugon-Moulin N, 2002 The estimation of population differentiation with microsatellite markers. Mol Ecol 11: 155–165. 11856418 10.1046/j.0962-1083.2001.01436.x

[26] Haubold B Hudson RR, 2000 LIAN 3.0: detecting linkage disequilibrium in multilocus data. Linkage analysis. Bioinformatics 16: 847–848. 11108709 10.1093/bioinformatics/16.9.847

[27] Havryliuk T Orjuela-Sanchez P Ferreira MU, 2008 *Plasmodium vivax*: microsatellite analysis of multiple-clone infections. Exp Parasitol 120: 330–336. 18801362 10.1016/j.exppara.2008.08.012

[28] Team RC, 2013 *R: A Language and Environment for Statistical Computing*. Vienna, Austria: R Foundation for Statistical Computing.

[29] Noviyanti R 2015 Contrasting transmission dynamics of co-endemic *Plasmodium vivax* and *P. falciparum*: implications for malaria control and elimination. PLoS Negl Trop Dis 9: e0003739. 25951184 10.1371/journal.pntd.0003739 PMC4423885

[30] Poirot E Skarbinski J Sinclair D Kachur SP Slutsker L Hwang J, 2013 Mass drug administration for malaria. Cochrane Database Syst Rev CD008846. 24318836 10.1002/14651858.CD008846.pub2 PMC4468927

[31] Marfurt J Smith TA Hastings IM Muller I Sie A Oa O Baisor M Reeder JC Beck HP Genton B, 2010 *Plasmodium falciparum* resistance to anti-malarial drugs in Papua New Guinea: evaluation of a community-based approach for the molecular monitoring of resistance. Malar J 9: 8. 20053293 10.1186/1475-2875-9-8 PMC2820042

[32] Ferreira MU Nair S Hyunh TV Kawamoto F Anderson TJ, 2002 Microsatellite characterization of *Plasmodium falciparum* from cerebral and uncomplicated malaria patients in southern Vietnam. J Clin Microbiol 40: 1854–1857. 11980977 10.1128/JCM.40.5.1854-1857.2002 PMC130917

[33] Pacheco MA Lopez-Perez M Vallejo AF Herrera S Arevalo-Herrera M Escalante AA, 2016 Multiplicity of infection and disease severity in *Plasmodium vivax*. PLoS Negl Trop Dis 10: e0004355. 26751811 10.1371/journal.pntd.0004355 PMC4709143

[34] Auburn S Barry AE, 2017 Dissecting malaria biology and epidemiology using population genetics and genomics. Int J Parasitol 47: 77–85. 27825828 10.1016/j.ijpara.2016.08.006

[35] Tripura R 2016 Persistent *Plasmodium falciparum* and *Plasmodium vivax* infections in a western Cambodian population: implications for prevention, treatment and elimination strategies. Malar J 15: 181. 27013512 10.1186/s12936-016-1224-7 PMC4806483

[36] Havryliuk T Ferreira MU, 2009 A closer look at multiple-clone *Plasmodium vivax* infections: detection methods, prevalence and consequences. Mem Inst Oswaldo Cruz 104: 67–73. 19274379 10.1590/s0074-02762009000100011

[37] Vafa M Troye-Blomberg M Anchang J Garcia A Migot-Nabias F, 2008 Multiplicity of *Plasmodium falciparum* infection in asymptomatic children in Senegal: relation to transmission, age and erythrocyte variants. Malar J 7: 17. 18215251 10.1186/1475-2875-7-17 PMC2267475

[38] Escalante AA 2015 Malaria molecular epidemiology: lessons from the International Centers of Excellence for Malaria Research Network. Am J Trop Med Hyg 93: 79–86. 26259945 10.4269/ajtmh.15-0005 PMC4574277

[39] Nkhoma SC Nair S Al-Saai S Ashley E McGready R Phyo AP Nosten F Anderson TJ, 2013 Population genetic correlates of declining transmission in a human pathogen. Mol Ecol 22: 273–285. 23121253 10.1111/mec.12099 PMC3537863

[40] Abdullah NR 2013 *Plasmodium vivax* population structure and transmission dynamics in Sabah Malaysia. PLoS One 8: e82553. 24358203 10.1371/journal.pone.0082553 PMC3866266

[41] Galatas B Bassat Q Mayor A, 2016 Malaria parasites in the asymptomatic: looking for the hay in the haystack. Trends Parasitol 32: 296–308. 26708404 10.1016/j.pt.2015.11.015

[42] Gatei W 2014 Genetic diversity of *Plasmodium falciparum* parasite by microsatellite markers after scale-up of insecticide-treated bed nets in western Kenya. Malar J 13 (*Suppl 1*): 495. 26651480 10.1186/s12936-015-1003-x PMC4675068

