# Supplementary material for: Passively versus Actively Detected Malaria: Similar Genetic Diversity but Different Complexity of Infection
Source: Am J Trop Med Hyg. 2017 Sep 5;97(6):1788–96. doi: 10.4269/ajtmh.17-0364 (PMC5790166; doi:10.4269/ajtmh.17-0364)
Supplement: Supplementary file 1 [file tpmd170364.SD1.pdf]

Supplemental Table 1

Marker diversity and genotyping success rate in *Plasmodium falciparum* and *Plasmodium vivax*

Species	Markers	CS H_E	ESS H_E	TES H_E	Failure, *N* (%)
P. vivax[75] (*N* = 477)	*MS12*	0.736	0.754	0.840	99 (20.8)
	pv3.27	0.868	0.897	0.845	88 (18.4)
	msp1f3	0.822	0.784	0.841	90 (18.9)
	MS10	0.897	0.900	0.896	97 (20.3)
	MS5	0.847	0.854	0.833	90 (18.9)
	MS1	0.779	0.747	0.843	102 (21.4)
	MS16	0.951	0.926	0.952	93 (19.5)
	MS20	0.892	0.893	0.917	125 (26.2)
P. falciparum[22] (*N* = 470)	*Poly-alpha*	0.748	0.596	0.627	57 (12.1)
	TA42	0.410	0.062	0.170	122 (26.0)
	TA81	0.827	0.783	0.703	88 (18.7)
	TA87	0.698	0.585	0.680	44 (9.4)
	ARAII	0.810	0.755	0.746	57 (12.1)
	PfPK2	0.733	0.693	0.319	52 (11.1)
	TA60	0.744	0.662	0.635	26 (5.5)
	TA1	0.706	0.601	0.418	118 (25.1)
	TA109	0.230	0	0.044	41 (8.7)

CS = community survey; ESS = ex vivo susceptibility study; H_E = expected heterozygosity; TES = treatment efficacy study; # Indicates the number of samples that failed all loci.

Supplemental Table 2

Temporal analysis of genetic diversity and within-host diversity

Sampling method	Year	Species	All cases	MOI,* Mean SD† (range)	Polyclonality, N (%)	H_E‡ Mean ±SD (range)	*Rs*§ Mean (SD)
Passively detected	2011	*P. falciparum*	11	1.1, 0.3 (1–2)	1 (9)	0.511 ± 0.3 (0–0.782)	2.571 (1.11)
		P. vivax	35	1.3, 0.5 (1–3)	8 (23)	0.841 ± 0.082 (0.716–0.959)	5.075 (1.20)
	2012	*P. falciparum*	36	1.1, 0.2 (1–2)	2 (6)	0.530 ± 0.307 (0–0.794)	2.831 (1.11)
		P. vivax	30	1.6, 0.8 (1–4)	13 (43)	0.846 ± 0.079 (0.720–0.929)	5.285 (0.91)
	2013	*P. falciparum*	13	1.3, 0.5 (1–2)	4 (31)	0.608 ± 0.278 (0–0.808)	3.328 (1.19)
		P. vivax	25	1.3, 0.5 (1–2)	7 (28)	0.840 ± 0.073 (0.747–0.913)	4.927 (0.90)
	2014	*P. falciparum*	34	1.2, 0.4 (1–2)	7 (21)	0.502 ± 0.283 (0–0.763)	2.672 (1.02)
		P. vivax	14	1.6, 0.6 (1–3)	7 (50)	0.861 ± 0.084 (0.714–0.967)	5.336 (1.23)
	2015	*P. falciparum*	37	1.1, 0.3 (1–2)	3 (8)	0.469 ± 0.246 (0.054–0.766)	2.690 (1.00)
		P. vivax	39	1.3, 0.5 (1–2)	12 (32)	0.870 ± 0.047 (0.820–0.956)	5.481 (0.70)
	2016	*P. falciparum*	8	1, NA	0	0.448 ± 0.311 (0–0.786)	2.778 (1.48)
		P. vivax	9	2, 1.0 (2–4)	6 (67)	0.881 ± 0.047 (0.806–0.944)	5.306 (0.88)
Actively detected	2013	*P. falciparum*	331	1.2 (1–3)	51 (19)	0.656 ± 0.2 (0.230–0.827)	7.112 (2.38)
		P. vivax	324	1.7 (1–4)	125 (52)	0.848 ± 0.069 (0.734–0.951)	13.00 (3.94)

* Multiplicity of infection.
† Standard deviation.
‡ Expected heterozygosity.
§ Allelic richness.
NA = not applicable.

Supplemental Table 3

Temporal analysis of linkage disequilibrium

Subgroup	All infections *N*	I_A^S	Low complexity *N*	I_A^S	Unique haplotypes *N*	I_A^S
P. falciparum						
2011	11	−0.0201^{NS}	11	−0.0201^{NS}	11	−0.0201^{NS}
2012	36	0.0833^{NS}	35	0.0901**	28	0.0346**
2013	13	0.0338^{NS}	10	0.0606*	13	0.0338*
2014	34	0.0314**	30	0.0243*	28	0.0147*
2015	37	0.1642**	35	0.1582**	25	0.0624**
P. vivax						
2011	35	0.0334*	29	0.0232^{NS}	25	0.0334*
2012	30	0.0015^{NS}	25	0.0092^{NS}	21	0.0015^{NS}
2013	28	−0.0046^{NS}	22	0.007^{NS}	23	−0.0046^{NS}
2014	14	0.0092^{NS}	9	−0.0323^{NS}	14	0.0092^{NS}
2015	37	0.0123^{NS}	30	0.0222*	30	−0.0038^{NS}

Only samples with no missing data were included in the analyses. * $P < 0.05$; ** $P < 0.01$; *** $P < 0.001$; NS = not significant.

Supplemental Table 4

Within-host and population diversity between adults and children with asymptomatic *Plasmodium falciparum and Plasmodium vivax* infections

	N	MOI Mean* (SD)	*P*†	Polyclonality *N* (%)	Chi	R_S‡ Mean (SD)	*P*†	H_E§ mean (SD)	*P*†	F_{ST}‖	*P* ± SD	F'_{ST}
P. falciparum												
All adults#	92	1.2 (0.4)	0.868	19 (20)	0.796	5.44 (1.92)	0.635	0.701 (0.169)	0.536	0.009	0.987 ± 0.0004	−0.031
All children**	90	1.2 (0.4)		21 (22)		5.66 (1.71)		0.675 (0.187)				
P. vivax												
All adults	57	1.6 (0.8)	0.242	25 (44)	0.157	10.83 (4.44)	0.52	0.829 (0.088)	0.429	−0.003	0.710 ± 0.0015	−0.020
All children	112	1.7 (0.8)		62 (55)		10.56 (3.77)		0.855 (0.066)				

MOI = multiplicity of infection.
* Standard deviation.
† Mann–Whitney *U* test.
‡ Allelic richness.
§ Expected heterozygosity.
‖ Fixation index.
¶ Standardized fixation index.
# Adults ≥ 15 years old.
** Children < 15 years old.

Supplemental Table 5

Within-host and population diversity between capillary and venous samples from asymptomatic *P. falciparum and P. vivax* infections

Subgroups	*N*	MOI Mean* (SD)	*P*†	Polyclonality N (%)	Chi	R_S‡ Mean (SD)	*P*†	H_E§ Mean (SD)	*P*†	F_{ST}‖	*P* ± SD	F'_{ST}¶
P. falciparum												
All capillary	182	1.1 (0.343)	0.122	39 (21)	0.124	6.00 (1.83)	0.081	0.687 (0.171)	0.113	0.026	0.002 ±0.0001	0.073
All venous	82	1.2 (0.428)		11 (13)		4.81 (1.90)		0.563 (0.283)				
P. vivax												
All capillary	169	1.7 (0.791)	0.346	87 (51)	0.837	12.85 (5.37)	0.809	0.846 (0.071)	0.959	0.008	0.017 ± 0.0004	0.048
All venous	68	1.6 (0.608)		34 (50)		12.88 (5.65)		0.848 (0.079)				

MOI = multiplicity of infection.
* Standard deviation.
† Mann–Whitney *U* test.
‡ Allelic richness.
§ Expected heterozygosity.
‖ Fixation index.
¶ Standardized fixation index.

Supplemental Table 6

Within-host diversity and stage synchronicity between *Plasmodium falciparum and Plasmodium vivax* sample sets

Sample sets	Synchronous*	Synchronous *N* (%)	MOI Mean† (SD)	Polyclonality *N* (%)	Total
P. falciparum					
CS	Yes	84 (89)	1.23 (0.42)	19 (23)	94
	No	10 (11)	1.27 (0.48)	3 (30)	
TES	Yes	44 (98)	1.07 (0.25)	3 (7)	45
	No	1 (2)	1.00 (0)	0 (0)	
ESS	Yes	94 (100)	1.15 (0.36)	14 (15)	94
	No	0 (0)	0 (0)	0 (0)	
All	Yes	222 (95)	1.16 (0.37)	36 (16)	233
	No	11 (5)	1.27 (0.47)	3 (27)	
P. vivax					
CS	Yes	9 (11)	1.80 (1.03)	4 (44)	81
	No	72 (89)	1.75 (0.82)	39 (54)	
TES	Yes	19 (44)	1.07 (0.25)	7 (37)	46
	No	27 (56)	1.52 (0.75)	11 (41)	
ESS	Yes	104 (100)	1.39 (0.61)	35 (34)	104
	No	0 (0)	0 (0)	0 (0)	
All	Yes	132 (57)	1.55 (0.73)	46 (35)	231
	No	99 (43)	1.69 (0.80)	50 (51)	

CS = community survey; ESS = ex vivo susceptibility study; MOI = multiplicity of infection; TES = treatment efficacy study.
* Ring stage > 70%.
† Standard deviation.
